# Vocation of Human Care and Soft Skills in Nursing and Physiotherapy Students: A Cross-Sectional Study

**DOI:** 10.3390/nursrep15020070

**Published:** 2025-02-15

**Authors:** Juan-Elicio Hernández-Xumet, Alfonso-Miguel García-Hernández, Jerónimo-Pedro Fernández-González, Cristo-Manuel Marrero-González

**Affiliations:** 1 Movement and Health Research Group, Departamento de Medicina Física y Farmacología, Facultad de Ciencias de la Salud, Universidad de La Laguna (ULL), 38200 La Laguna, Spain; jfernago@ull.edu.es (J.-P.F.-G.); cmarrerg@ull.edu.es (C.-M.M.-G.); 2 Hospital Universitario Nuestra Señora de Candelaria, Servicio Canario de la Salud, 38010 Santa Cruz de Tenerife, Spain; 3 Departamento de Enfermería, Facultad de Ciencias de la Salud, Universidad de La Laguna (ULL), 38200 La Laguna, Spain; almigar@ull.edu.es; 4 Gerencia de Atención Primaria de Tenerife, Servicio Canario de la Salud, 38004 Santa Cruz de Tenerife, Spain

**Keywords:** students, nursing, physical therapy, empathy, assertiveness, vocation for care

## Abstract

**Background:** Empathy and assertiveness are two essential soft skills for health professionals such as nurses or physiotherapists. Both professions are characterised by their contribution to preserving and improving health and human care, and for their vocation of service. One of the achievements of the training is the voluntary, conscious, and individually inspired career choice. **Objectives:** This study aimed to evaluate the empathy, assertiveness, and care vocational levels among undergraduate students enrolled in Nursing and Physiotherapy Degree programmes and to investigate the relationship between these soft skills and the care vocation. **Methods:** A cross-sectional study of nursing and physiotherapy students was conducted in the 2023/2024 academic year. The Interpersonal Reactivity Index scales for empathy, the Rathus test for assertiveness, and the “Vocation of Service for Human Care” questionnaire were used as study tools. Finally, 226 nursing and physiotherapy students participated after providing informed consent, excluding those on national or international exchange programmes. **Results:** The empathy, assertiveness, and vocation of service for human care of the students was found to be acceptable. Significant differences were observed according to the gender variable or career among the students, with female students presenting better results in empathy subscales or vocation (*p* < 0.05). Students who were working or had clinical experience in other professions scored lower on the empathy personal distress subscale (*p* < 0.05). **Conclusions:** Both nursing and physiotherapy students demonstrate satisfactory levels of empathy, assertiveness, and vocation for human care. However, gender and previous work experience notably influence these scores. It would be beneficial to conduct long-term studies with educational interventions to train and develop empathy, assertiveness, and vocation for human care.

## 1. Introduction

Vocation plays a crucial role in professional practice, particularly in healthcare. While not innate, it involves aptitudes and sensitivity to others’ suffering alongside key virtues such as compassion, discernment, and trust, which underpin ethical professional development [1,2]. Advances in science and globalisation have reshaped clinical relationships, emphasising holistic care and the relational nature of humans. Individuals exist within networks of interactions driven by lifelong needs, and the ethics of care prioritise these relationships over universal principles, integrating rationality, affect, and emotion [3].

A vocation for care is a personal and deep inclination that drives a person to work for the care and well-being of others. In contrast to professionalism, which refers to an ethical and responsible approach to the practice of a profession, a vocation for care goes beyond meeting the technical or regulatory standards of a profession.

Martins et al. [4] argue for integrating ethics education into medical and nursing curricula, considering it as vital as technical skills. Marqués-Sule et al. advocate for embedding ethics throughout physiotherapy training [5]. This ensures that students develop competencies to navigate clinical scenarios responsibly, emphasising beneficence, non-maleficence, and patient autonomy.

Empathy, altruism, and compassion form a foundational triad in healthcare. These traits require academic reinforcement to prepare students for human-centred care [6,7]. Training empathy enhances students’ compassionate capacity [8] and fosters a secure care environment [9]. Mármol-López et al. [10] propose that ethics education for physiotherapists should emphasise attitude development alongside technical and bioethical training.

### 1.1. Vocation for Care

Anaya-Requejo [11] finds no significant sociodemographic factors influencing nursing vocation, while Limanta-Barrios [12] identifies a link between socio-personal variables and vocational interest. Arenas-Ramírez et al. [13] state that vocational levels among nursing students vary by semester and correlate with happiness, impacting professional performance. Similarly, Van den Boogaard et al. [14] observe an initial enthusiasm for care that wanes during training, stressing the need for psychological interventions such as Psychological First Aid (PFA) to boost resilience and self-efficacy [15].

The authors advocate continued research into vocational dynamics for improving healthcare quality. Akhter et al. [16] emphasise educators’ roles in fostering vocation, calling for a shift in training to nurture critical and ethical thinking alongside technical skills [17].

Fuente-Vidal et al. [18] point to altruism as a key motivator for physiotherapy students, with gendered differences: men focus on sports-related aspects, while women prioritise altruism and scientific interest.

In the health professions (nursing, physiotherapy, or medicine), these elements are particularly relevant since a vocation for care would not only improve the relationship with patients, but also enrich the professional practice by integrating a more human and transformative dimension into the care provided.

### 1.2. The Role of Soft Skills: Empathy and Assertiveness

Empathy training is critical in healthcare education, as highlighted by Jiao et al. [19], who highlight its role in improving patient care. Moudatsou et al. [20] link enhanced empathy to positive nurse–patient interactions, advocating targeted training for students. Lopes and Nihei [21] caution against the emotional toll of caregiving, with burnout risks necessitating resilience-building efforts [22].

Immersive simulations and experiential learning emerge as effective empathy training methods, improving communication, self-efficacy, and emotional engagement [23,24,25,26,27,28,29,30,31]. Rodríguez-Nogueira et al. [32] advocate service learning to develop socioemotional skills and reduce distress among physiotherapy students. Additionally, incorporating humanities into the curricula—such as art and literature—can enrich empathic development, as reported by Blanton et al. [33].

Clinical internships further enhance students’ communication and empathy skills [34]. Yu et al. [35] report higher empathy levels among interns, though González-Serna et al. [36] observe declines in empathy during clinical practice. Longitudinal studies are needed to explore these dynamics fully. Structured reflective activities during placements, as suggested by Imperato and Strano-Paul [37], can strengthen empathic thinking.

Percy and Richardson [38] assert that empathy and compassion must be integral to training. Güven et al. [39] add that assertiveness complements empathy, promoting altruistic thinking and emotional intelligence, thus improving the quality of care [40,41].

### 1.3. The Importance of Ethical and Assertive Training

Aguilar-Rodríguez et al. [42] argue that ethical training is crucial for identifying bioethical dilemmas in patient care. Developing assertiveness alongside empathy equips professionals to navigate interpersonal challenges and conflicts [43]. Luna et al. [44] highlight a link between assertiveness, self-esteem, and mental well-being.

Assertiveness training fosters better communication, job satisfaction, and patient care [45]. Ibrahim [46] finds higher assertiveness among advanced nursing students, linked to psychological empowerment and family income.

Integrating vocational, ethical, and soft skills training into healthcare education ensures that future professionals are not only technically competent, but also emotionally and ethically prepared to deliver quality care. By fostering empathy, compassion, and assertiveness through structured education, immersive experiences, and reflective practices, healthcare students can develop the competencies necessary to navigate the complexities of modern clinical environments.

## 2. Materials and Methods

### 2.1. Research Objectives

The objective of the present study is to assess the state of vocational care and the empathic and assertive levels of nursing and physiotherapy students at the University of La Laguna, to study the differences and similarities between these types of students’ scores and investigate the relationship between vocation for care and other soft skills such as empathy and assertiveness in nursing and physiotherapy students.

### 2.2. Research Design and Participants

An observational, descriptive, and cross-sectional study design was proposed following the STROBE guidelines.

### 2.3. Research Design

The research team contacted professors and student representatives from the degree course to ensure maximum participation from physiotherapy students. Students from each academic year were convened for a face-to-face meeting where the purpose of the research was explained, as well as the steps to follow to participate in the study; the aim in this first phase of the study was to provide information about the study.

**Figure 1:**
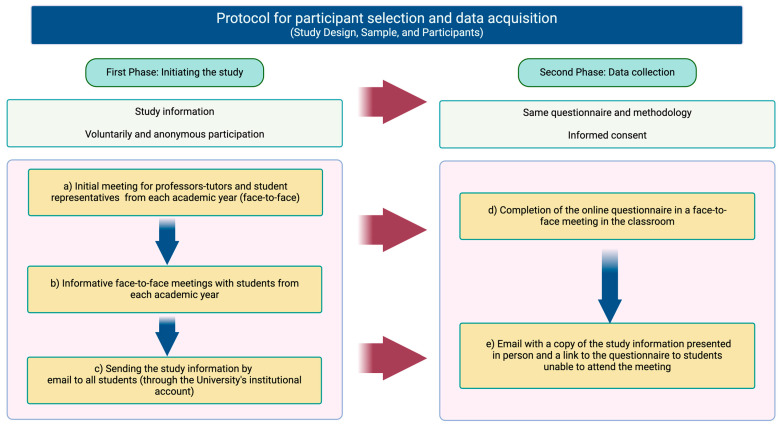
The diagram illustrates the process of selecting students and how the data were obtained. Created with BioRender.com.

In addition, all of the students were invited via email to complete the questionnaire in a face-to-face meeting prior to the information day of the study. Subsequently, all students unable to attend the meetings (information or questionnaire) were sent an email with a copy of the study information presented in person and a link to the questionnaire that they could fill in voluntarily and anonymously, and informed consent was also obtained from the participants. The students were free to ask any questions before completing the questionnaire. The questionnaire was available for four weeks in November and December 2023 (from 20 November to 15 December). The questionnaire format was the same for all study participants; they always had to use the institutional account to access the questionnaire. The questionnaire was designed with a configuration that limited each student’s access to a single opportunity to complete it. This restriction was previously communicated to the students through the previous information process, ensuring that each participant could complete the activity only once. This approach was adopted to help control the possible duplication of responses and to preserve the integrity of the data collected. For the data collection and information protocol, see Figure 1.

### 2.4. Participants and Place of Study

The sample consisted of undergraduate nursing and physiotherapy students of the University of La Laguna. A total of 226 students, aged over 18, were recruited from September to December 2023. There were 93 nursing students and 133 physiotherapy students. All participants were between 18 and 58 years old (M = 23.59; SD = 8.51).

The gender distribution of the sample was 71 men (31.40%) and 155 women (68.60%). The academic year distribution of students was 63 first-year (63/160), 63 second-year (63/160), 53 third-year (53/160) and 47 fourth-year students (47/160). The distribution of nursing and physiotherapy students was 133 physiotherapy students and 93 nursing students. Regarding the employment status of the students, 22.60% (51/226) were working, and 19.00% (43/226) of the students had work experience in health sciences.

### 2.5. Inclusion and Exclusion Criteria

All enrolled participants were informed of the study’s purpose and procedures and provided their written informed consent. The inclusion criteria were (1) university nursing and physiotherapy students, and (2) students who consented to participate in the study with full knowledge of its purpose and content. Students had to meet both criteria to be included in the study.

The exclusion criterion was external university students on a national or international exchange programme, that is, Erasmus, Sicue, or similar. The Erasmus programme is a student exchange programme between European universities, and the Sicue programme is an exchange programme similar to Erasmus but between universities in Spain. In these programmes, students spend 6–12 months at the university and have different academic and professional backgrounds to local students.

### 2.6. Outcome Measures

#### 2.6.1. Instrument for Measuring Empathy: Interpersonal Reactivity Index (IRI)

The IRI provides separate assessments of cognitive and emotional processes. This index was initially developed by Davis [47,48]. It allows empathy to be viewed as a set of four factors rather than as a unidimensional concept. It is an easy-to-use scale consisting of 28 items divided into four subscales measuring four dimensions of the overall concept of empathy: perspective taking (PT), fantasy (FS), empathic concern (EC), and personal distress (PD), each with seven items.

The PT and FS subscales assess cognitive processes. The PT score indicates the person’s spontaneous attempts to consider another person’s perspective in real-life situations.

The FS subscale measures the tendency to identify with film and literary characters, i.e., the person’s imaginative ability to place themselves in fictional situations.

The EC and PD subscales measure people’s emotional reactions to others’ negative experiences. The EC measures feelings of sympathy, concern, and care in the face of others’ discomfort. The PD measures feelings of anxiety and discomfort when perceiving the negative experiences of others.

The 28 items consist of a Likert-type response scale with five options according to the degree to which the statement describes the respondent (0 = does not describe me well; 1 = describes me a little; 2 = describes me well; 3 = describes me fairly well; and 4 = describes me very well) [47,48,49,50].

The IRI—Spanish version is one of the most widely used self-reporting measures to assess students’ empathy [49,50].

#### 2.6.2. Instrument for Measuring Assertiveness: Rathus Scale

The Rathus Assertiveness Scale (RAS) was designed to measure a person’s level of assertiveness. It is also an instrument for measuring behavioural change in assertion training. The RAS was developed in 1973 by Spencer Rathus [51]. The RAS consists of 30 items (including 16 inverted items) with a 7-point Likert scale scored from −3 (very uncharacteristic of me) to 3 (very characteristic of me).

Leon-Madrigal and Vargas Halabí [52] validated the RAS scale in Spanish and subsequently revised it [53].

#### 2.6.3. Instrument to Measure the Vocation for Healthcare

The instrument “Vocation of Service for Human Care” was created by Antonio-González [54] to provide a valid and specific instrument to determine the vocational level for human care. This questionnaire “will identify students who have a low service vocation for human care and, therefore, to carry out interventions to increase the service vocation for human care and, therefore, to reduce dropout” [55]. The instrument considers patterns of knowledge, confidence in the vocation for care, and values and satisfaction with the chosen career. It consists of 23 items constructed on a Likert scale. Each item is answered with 1 = never, 2 = rarely, 3 = sometimes, 4 = almost always, and 5 = always [54,55].

The definition of the primary variable of this questionnaire, the vocation of service for human care, is the inclination or sense of inspiration that the student has to dedicate care to the individual, family, or community. This instrument consists of three dimensions: one called “inclination towards health care”, a second called “self-efficacy in health care”, and a third called the “axiological component”. The first dimension can be defined as the ease with which the student can provide care to healthy and sick people in community and clinical settings (items 3, 5, 7, 10, 12, 16, and 19). The second is defined as the student’s confidence in providing care to healthy and sick people (items 1, 2, 4, 9, 11, 13, 15, 17, and 20). Finally, the axiological component is the set of ethical and moral values that the student has in their personal, professional, and social life (items 6, 8, 14, 21, 22, and 23) [54].

### 2.7. Statistical Analysis

Data management and analysis were performed using SPSS 26.0 (IBM, 2019) and Jamovi version 2.3.17 (Project, 2023). Descriptive analyses were performed, and mixed ANOVAs were conducted with the intragroup factor (pre/post) and the intergroup factor of gender (male/female) to test whether gender or clinical practice affected empathy or assertiveness. The relationship between empathy and assertiveness scales was analysed using Pearson’s correlation analysis. A *p*-value ≤ 0.05 was considered statistically significant.

### 2.8. Ethical Considerations

The University Research Ethics Committee (CEIBA-ULL) approved the study with code CEIBA2022-3133.

All students were recruited voluntarily and were free to withdraw from the study at any time. No participants were coerced or pressured to complete the survey, and they provided their consent for participation in the study.

## 3. Results

### 3.1. Sample Description

A total of 226 students participated in the study, and 226 questionnaires were received (all questionnaires completed by students were valid; there were no partially completed questionnaires or missing data). The sample represented 35.31% of the total population of nursing and physiotherapy students at the university (226/640 students).

#### 3.1.1. Interpersonal Reactivity Index (IRI—Spanish Version)

The university nursing and physiotherapy students obtained an overall empathy score on the IRI subscales of perspective taking (PT) (M = 27.71; SD = 4.12; Cronbach’s α = 0.70), empathic concern (EC) (M = 27.92; SD = 3.81; Cronbach’s α = 0.82), fantasy (FS) (M = 23.60; SD = 5.84; Cronbach’s α = 0.79), and personal distress (PD) (M = 15.76; SD = 4.40; Cronbach’s α = 0.69). The results from each subscale for the nursing and physiotherapy students, by gender and academic year, are shown in Table 1.

#### 3.1.2. The Rathus Assertiveness Scale (RAS—Spanish Version)

The nursing and physiotherapy students obtained a global RAS score of −2.16 (SD = 25.09; Cronbach’s α = 0.86). Here, 10.20% of students (23/226) achieved a score of “very assertive”, 75.20% (170/226) “acceptable assertiveness”, and 14.60% (33/226) “slightly assertive.” The RAS scores for the different assertiveness results grouped by academic year and gender are shown in Table 2.

#### 3.1.3. The “Vocation of Service for Human Care” Questionnaire

The nursing and physiotherapy students received a global vocation score of 79.37 points for “good vocation”; between 50 and 75 is a regular vocation, and above 75 is considered good (SD = 11.84; Cronbach’s α = 0.88). In addition, 37.60% (85/226) obtained “regular vocation”, and 62.40% (141/226) obtained “good vocation” (no student’s score indicated “low vocation” or “very low vocation”). The vocation scores by academic year and gender are shown in Table 3.

### 3.2. Inferential Analysis

#### 3.2.1. Empathy

Significant differences were found in the multivariate analysis in relation to gender (F_4,219_ = 7.192; *p* < 0.001; *η*^2^ = 0.116) and the specific career of the students (F_4,219_ = 3.087; *p* = 0.017; *η*^2^ = 0.053). No statistical significance was found for the interaction between gender and the student’s career (F_4, 219_ = 0.864; *p* = 0.486).

**Figure 2:**
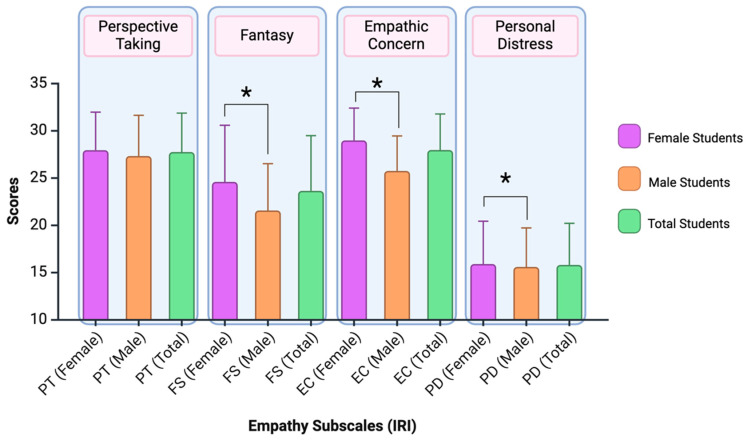
The figure shows the results for male and female students on each empathy subscale (PT = perspective taking; FS = fantasy; EC = empathic concern; PD = personal distress). Subscales with significant differences (*p* < 0.05) are marked with *. Created with BioRender.com.

Empathy subscales were analysed while considering interactions, and significant differences were found in three empathy subscales concerning the gender variable (fantasy, empathic concern, and personal distress; see Figure 2). Females scored higher than males in the fantasy subscale (F_3,222_ = 11.931; *p* = 0.001; *η*^2^ = 0.051), the empathic concern subscale (F_3,222_ = 25.070; *p* < 0.001; *η*^2^ = 0.101), and the personal distress subscale (F_3,222_ = 3.943; *p* = 0.048; *η*^2^ = 0.017).

**Figure 3:**
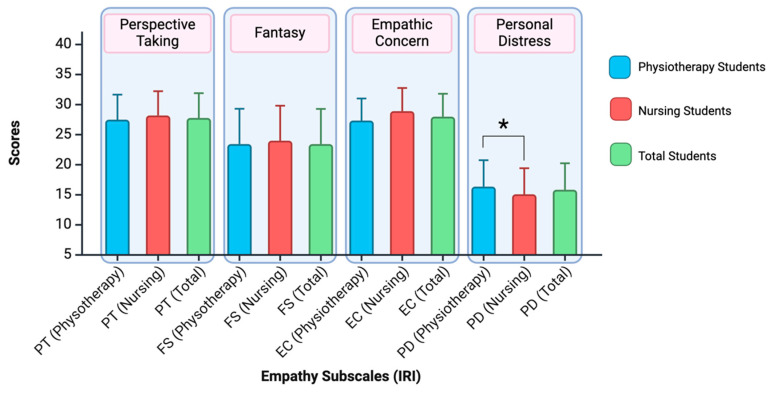
The figure shows the results for nursing and physiotherapy students on each empathy subscale (PT = perspective taking; FS = fantasy; EC = empathic concern; PD = personal distress). Subscales with significant differences (*p* < 0.05) are marked with *. Created with BioRender.com.

Significant differences were found in the empathy subscale PD concerning the specific career of the students (see Figure 3). The physiotherapy students scored higher than nursing students for the personal distress subscale (F_3,222_ = 9.402; *p* = 0.002; *η*^2^ = 0.041).

Significant differences were also found in the FS and PD empathy subscales concerning questions about working while studying (F_4,219_ = 2.527; *p* = 0.042; *η*^2^ = 0.044). Students concurrently studying with a job had lower scores on the FS (F_3, 222_ = 3.913; *p* = 0.049; *η*^2^ = 0.017) and PD (F_3,222_ = 6.768; *p* = 0.010; *η*^2^ = 0.030) empathy subscales.

No significant differences were found in the empathy subscales concerning academic year (PT—F_3,222_ = 0.059; *p* = 0.981; *η*^2^ = 0.001/EC—F_3,222_ = 1.462; *p* = 0.226; *η*^2^ = 0.019/FS—F_3,222_ = 0.982; *p* = 0.402; *η*^2^ = 0.013/PD—F_3,222_ = 1.770; *p* = 0.154; *η*^2^ = 0.023).

#### 3.2.2. Assertiveness

**Figure 4:**
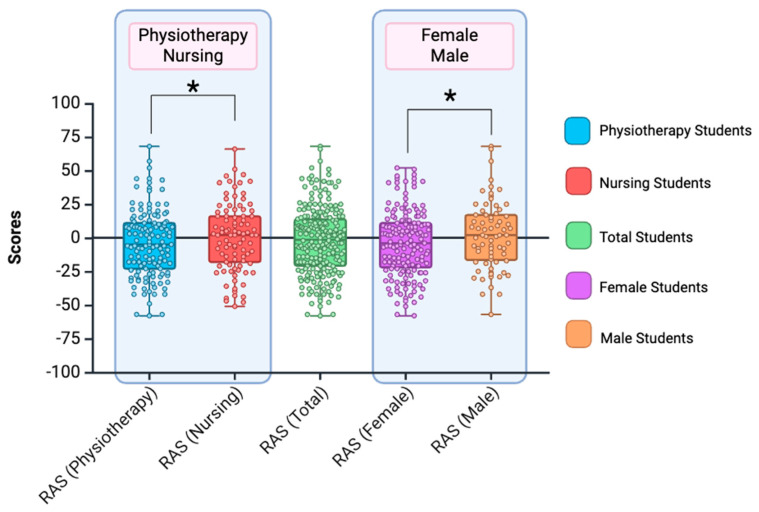
The figure shows the results for male, female, nursing, and physiotherapy students for assertiveness (RAS). Significant differences (*p* < 0.05) are marked with *. Created with BioRender.com.

Significant differences were found in the multivariate analysis for gender (F_3,222_ = 6.356; *p* = 0.012; *η*^2^ = 0.028) and the specific career of the students (F_3,222_ = 5.481; *p* = 0.020; *η*^2^ = 0.024). Males scored higher than females in assertiveness. Nursing students scored higher in assertiveness than physiotherapy students (see Figure 4). No statistical significance was found for the interaction between gender and the student’s career (F_3,222_ = 1.487; *p* = 0.224).

Significant differences were also found in assertiveness regarding questions about working while studying (F_4,219_ = 7.721; *p* = 0.006; *η*^2^ = 0.034). Students concurrently studying with a job had lower scores for assertiveness.

No significant differences were found in assertiveness concerning academic year (F_3,222_ = 1.189; *p* = 0.315; *η*^2^ = 0.016).

#### 3.2.3. Vocation of Service for Human Care

**Figure 5:**
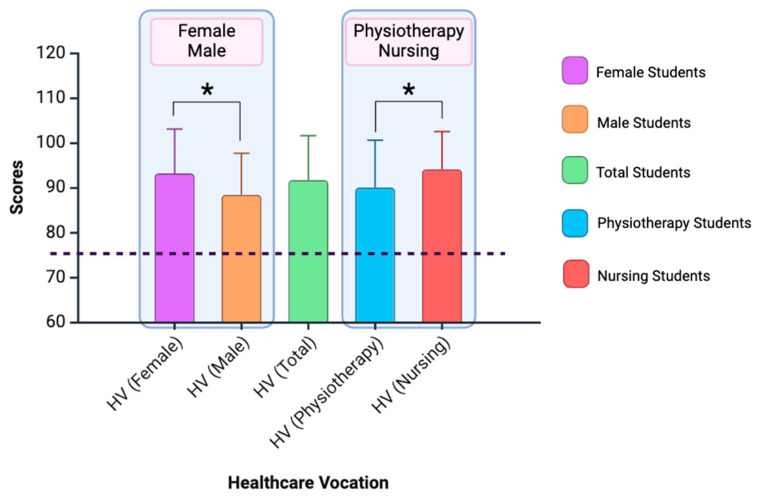
The figure shows the results for male, female, nursing and physiotherapy students on healthcare vocation (HV). Significant differences (*p* < 0.05) are marked with *. Created with BioRender.com.

Significant differences were found in the multivariate analysis for gender (F_3,220_ = 7.203; *p* < 0.001; *η*^2^ = 0.089) and the specific career of the students (F_3,220_ = 3.477; *p* = 0.017; *η*^2^ = 0.045). No statistical significance was found for the interaction between gender and the student’s career (F_3,220_ = 0.980; *p* = 0.403). See Figure 5.

The overall vocation score, as well as the three dimensions of the scale, were analysed while considering interactions, and significant differences were found concerning the gender variable (overall result and dimension 1). Females scored higher than males in terms of vocation (F_3,222_ = 13.114; *p* = 0.001; *η*^2^ = 0.151). Females scored higher on dimension 1 (F_3,222_ = 10.231; *p* = 0.002; *η*^2^ = 0.044). Differences in vocation were also found between nursing students and physiotherapy students (overall result and dimensions 1 and 2). Nursing students scored higher than physiotherapy students in terms of vocation (F_3,222_ = 9.134; *p* = 0.024; *η*^2^ = 0.076). Nursing students scored higher than physiotherapy students in dimension 1 (F_3,222_ = 8.558; *p* = 0.004; *η*^2^ = 0.037) and dimension 2 (F_3,222_ = 6.043; *p* = 0.015; *η*^2^ = 0.027).

No significant differences were found for vocation concerning questions about working while studying (F_4,222_ = 0.146; *p* = 0.703).

No significant differences were found for vocation concerning academic year (F_3,222_ = 1.114; *p* = 0.344; *η*^2^ = 0.015).

#### 3.2.4. Correlation Analysis

The perspective taking and fantasy empathy subscales showed a significant positive correlation with another empathy subscale: empathic concern. The perspective taking subscale had a positive correlation with empathic concern (r[226] = 0.306; *p* < 0.001). The fantasy subscale also had a positive correlation with empathic concern (r[226] = 0.442; *p* < 0.001).

The perspective taking subscale showed a significant positive correlation with vocation (overall—r[226] = 0.252; *p* < 0.001), and vocation dimension 1 (r[226] = 0.289; *p* < 0.001)

The personal distress empathy subscale showed a significant negative correlation with RAS—assertiveness (r[226] = −0.317; *p* < 0.001). The PD subscale also showed a significant negative correlation with vocation dimension 2 (r[226] = −0.260; *p* < 0.001).

The empathic concern empathy subscale showed a significant correlation with vocation (overall—r[226] = 0.351; *p* < 0.001), vocation dimension 1 (r[226] = 0.461; *p* < 0.001), and vocation dimension 3 (r[226] = 0.346; *p* < 0.001).

The results of the correlational analysis are shown in Table 4.

## 4. Discussion

The training of health professionals requires individuals with a vocation for service and a positive attitude who respond efficiently, empathetically, assertively, and humanistically to the demands of caring for life and restoring a person’s health [56,57,58]. Consequently, selecting a career in nursing or physiotherapy necessitates the possession of personal attributes that extend beyond the purely scientific and technical. These are highly humanistic professions in which professionals are dedicated to their social function. They are essential in health services, and the objectives focus on the prevention, promotion, and rehabilitation of health [57,58].

The present study highlights that nursing and physiotherapy students exhibit acceptable levels of empathy, assertiveness, and vocation for care. It is evident that, although the scale for measuring the “Vocation of Service for Human Care” was designed for nursing, physiotherapy students demonstrated comparable results of an acceptable level on this scale to nursing students. According to the findings of Antonio-González et al. [54,55], results exceeding a score of 76 points are indicative of a positive vocation. Among the results for the physiotherapy students, 92% achieved scores above this threshold, with none obtaining scores considered low or very low (below 50 points). Consequently, educators need to emphasise the importance of critical and ethical thinking in nursing and physiotherapy education.

Furthermore, physiotherapy students pursue these studies to provide assistance and care to others [17,18]. We concur with the author Anaya-Requejo [11] in that our findings revealed no correlation between sociodemographic variables and the decision to pursue a career in nursing. The student population across both degree programmes is sociodemographically diverse. However, as evidenced by the findings of Arenas-Ramírez et al. [13], it is important to monitor the levels of vocation displayed by students as they progress through their training, as these can fluctuate.

Regarding gender, females had higher scores in FS and EC than males, and males had higher scores in PD. This phenomenon is inferentially related to two of the three dimensions of the vocation of care: the inclination towards healthcare (dimension 1) and the axiological component (dimension 3).

A comparison of the scores of nursing and physiotherapy students revealed that nursing students tended to perform better than physiotherapy students on the EC empathy subscale and the vocation scale. However, physiotherapy students scored higher than nursing students on the PD subscale.

The results suggest that a variety of educational approaches may be beneficial. There is a clear need for targeted pedagogical interventions that enhance empathy and reduce emotional stress in both groups. These interventions could help promote the balanced development of the caring vocation and strengthen the quality of care offered to patients.

It is important to note that students who have a job in health science areas while studying for their degree in nursing or physiotherapy tended to obtain lower scores in the subscales of empathy (EC and FS) and vocation. However, they tended to obtain better scores in assertiveness (RAS) and higher scores in personal distress (PD). This phenomenon was also demonstrated in the study conducted by Hernández-Xumet et al. [59], in which students with previous experience in other health sciences exhibited higher scores in RAS and PD. In the present study, those with a job or professional experience in other areas or professions of health sciences exhibited the same phenomenon, with a significant proximity to dimension 2 of vocation for care (service self-efficacy). This suggests that although work experience strengthens their confidence and interpersonal skills, it may also potentially result in emotional fatigue or desensitisation, which impacts their vocation for care. Therefore, training programmes must consider incorporating strategies to balance these effects, such as training in stress management, resilience building, and empathy building. This could ensure that practical training is complemented by the emotional and vocational strengthening necessary for providing holistic and humane care.

The results of the present study demonstrate a correlation between the IRI subscales (PT, EC) and the three dimensions of vocation for care. Regarding the IRI FS subscale, a correlation was observed between EC and PD. The EC subscale correlated with dimensions 1 and 3 of vocation for care, while EC demonstrated an inverse correlation with RAS. A similar inverse correlation was observed with the PD subscale, which exhibited an inverse correlation between PD and the three dimensions of the vocation for care.

Individuals with high perspective taking (PT) levels are more likely to volunteer in a hospital or clinic. This is due to their enhanced capacity to comprehend the needs of patients and how they can assist them. Furthermore, such an individual is more likely to exhibit a robust ethical and moral compass, as they are better able to comprehend the needs of others and the impact of their actions on them.

Individuals with high levels of empathic concern (EC) are more likely to pursue careers in nursing or medicine. This is due to their strong compassion for the suffering of others and their desire to help them feel better. They are also more likely to act compassionately and altruistically, as they feel a strong connection with others and want to help them feel better.

The findings tentatively suggest that fostering emotional empathy (EC) and perspective taking (PT) as pillars for the development of the caring vocation while reducing personal distress (PD) and strengthening balanced assertiveness (RAS) could be beneficial for improving both the vocation and practice of caring in health science students. This implies the need for educational interventions that promote regulated emotional skills, such as resilience and assertive communication, without compromising empathic sensitivity.

### 4.1. Inferential Study

The findings consistently demonstrated that female students exhibited higher levels of empathy, which aligns with the results reported by Moreno-Segura et al. [60]. These results highlight the acceptable ethical and moral sensitivity demonstrated by physiotherapists. Furthermore, the study indicates that female participants exhibited higher levels of empathy than their male counterparts. It is, therefore, essential to understand these characteristics to design ethical training programmes that will enhance clinical care. As Marqués-Sule et al. [61] emphasise, future physiotherapists recognise the importance of ethics training in their professional development, as ethical awareness directly impacts clinical safety—a fundamental aspect of healthcare practice [1].

A comparable phenomenon can be observed in the field of nursing. Deng et al. [62] found that female nursing students exhibited higher levels of emotional intelligence than their male counterparts. These findings suggest that educators should address gender differences in empathy, emotional intelligence, and problem-solving skills. Women with higher empathy scores may feel more confident in their caregiving abilities, better understand patients’ needs, and provide more personalised care. Fostering inclusive learning environments that value diversity and cultivate students’ unique talents is essential to optimise these strengths.

Similarly, González-Serna et al. [36] reported higher empathy scores among female nursing students compared to males, a trend that was also observed among physiotherapy students by Hernández-Xumet et al. [59]. The data demonstrate that female students consistently show superior performance in empathy-related dimensions pertinent to health vocations in comparison to their male counterparts. This may indicate a greater propensity towards pursuing health-related careers within this cohort.

To illustrate the above, female physiotherapy students attained markedly higher scores than their male counterparts on the fantasy (FS) subscale, which assesses the capacity to identify with fictional characters and comprehend their emotional states. This indicates a greater capacity for emotional connection and empathy in hypothetical situations. A comparable pattern is observed in the empathic concern (EC) dimension, where females exhibited stronger compassion and emotional responsiveness to others’ needs and discomforts. These findings suggest a stronger tendency among female students to effectively understand and address others’ emotional experiences.

The observed differences in empathy and interpersonal skills between men and women in physiotherapy and nursing studies are multifactorial. Biological, socio-cultural, educational, and personal factors all influence these differences. From an evolutionary perspective, it is clear that women have developed more finely tuned skills in empathy and caring due to their historical role in nurturing and maintaining social relationships. This is reflected in contemporary contexts such as health professional education. Gender roles and social expectations instilled in childhood undoubtedly play a significant role, as people are often socialised to develop different skills. The social perception of health professions as “caring careers” undoubtedly attracts women with a predisposition towards empathy and interpersonal sensitivity, creating a selection bias reflected in the observed gender differences.

Soft skills training, such as empathy, should be structured to resonate more with women’s typical learning styles, encouraging their development and expression. We can reduce the observed differences by implementing methods that align with men’s learning styles. An educational environment that values empathic strengths and analytical skills benefits all students. By creating learning spaces that value the diversity of individual skills and talents, we can foster a more equitable approach to interpersonal skills development, benefiting both men and women. This approach optimises the training of future health professionals and promotes more equitable and high-quality care for patients.

### 4.2. Correlational Study

The correlational study used the four IRI subscales to assess the dimensions of empathy. The PT and FS subscales measure cognitive aspects such as considering another person’s perspective and imagining oneself in hypothetical situations. In contrast, EC and PD measure emotional responses to negative experiences: EC focuses on other-oriented feelings, while PD assesses self-oriented fear and discomfort. Notably, an inverse correlation was observed between PD and assertiveness (RAS), suggesting that students with higher assertiveness scores experience less personal distress (PD). This is consistent with Hernández-Xumet et al. [63], who reported similar findings.

Delgado et al. [64] examined the relationship between burnout and empathy in healthcare professionals. They found that perspective taking (PT) and empathic concern (EC) were positively correlated with mental state inference, whereas personal distress (PD) was associated with emotional exhaustion and depersonalisation. In line with this, the present study found low assertiveness correlated with high PD and low caregiving vocation, consistent with Hernández-Xumet et al. [63]. In addition, Luna et al. [44] found that nursing students with low assertiveness were more prone to anxiety and depression.

Addressing students’ emotional well-being is crucial, as burnout may affect their future nursing abilities. Carvalho et al. [65] stated that emotional intelligence supports the well-being of medical, nursing, and physiotherapy students, preventing burnout and enhancing patient care. Lee et al. [66] suggested that self-awareness is critical to developing empathic skills and improving satisfaction in clinical practice. Hernández-Xumet et al. [63] observed decreased empathy and assertiveness in fourth-year physiotherapy students after clinical practice. Assertiveness plays an essential role in healthcare. Students with higher levels of assertiveness are better communicators and more ethical and effective patient rights advocates, making them less likely to tolerate unethical practices or malpractice.

The relationship between assertiveness and the personal distress (PD) subscale indicates that more assertive students experience a reduction in anxiety or discomfort when observing negative situations. This is an ability to effectively manage personal emotions, which prevents them from interfering with patient care. In this context, assertiveness is an emotional regulation tool, enabling students to maintain objectivity without becoming emotionally desensitised. Students with higher levels of assertiveness can cultivate feelings of other-oriented empathy (EC) without experiencing the emotional exhaustion associated with PD. This is of particular importance in the context of healthcare professions, where excessive PD has the potential to contribute to burnout and emotional exhaustion.

There is a significant correlation between emotional exhaustion and depersonalisation, which are both associated with high levels of PD and low levels of CE. This may be explained by the accumulation of emotional burden in clinical settings, which affects the capacity of professionals to maintain balanced, patient-centred empathy. In this context, promoting assertiveness in students is an effective strategy for mitigating burnout, as it allows them to set clear boundaries and healthily manage conflict.

The data demonstrate that the relationship between empathy, assertiveness, and emotional well-being is intricate but pivotal in training healthcare professionals. Promoting assertiveness and self-awareness while attenuating the effects of burnout and enhancing interpersonal skills can be an efficacious strategy to optimise students’ training experience and the quality of care they will provide in the future.

The development of specific programmes focusing on assertiveness could improve communication between students, patients, and other health professionals. These skills not only promote more effective interactions, but also help students cope better with emotionally challenging situations. Similarly, designing more humane clinical practices, including emotional support for students, can prevent burnout associated with exposure to high-stress situations. In addition, encouraging reflection on clinical experiences can strengthen empathy and assertiveness skills.

### 4.3. Future Lines of Research

In light of the findings, the scope of research should extend beyond descriptive observational studies to encompass interventions aimed at enhancing or elucidating the development of a caring vocation among students. This is in line with the observations made by Arda Sürücü et al. [67], who posit that nursing students’ negative perceptions of cancer diminish as their empathic abilities improve. It is recommended that undergraduate training should not only include clinical training, but also empathic training.

Longitudinal studies help observe and monitor whether empathic development, for example, declines during students’ training. Yucel [68] observed a decline in empathy among fourth-year students in a longitudinal study with physiotherapy students. This decline was associated with clinical practice and academic courses. The author found that first-year students exhibited higher levels of empathy. Longitudinal studies involving educational interventions are highly beneficial. Consequently, Yang et al. [69] propose that longitudinal studies should be conducted to examine empathic development in nursing students. Educational interventions designed to foster empathy have been demonstrated to be effective at the experimental level and should be further integrated into undergraduate education.

As argued by Unal et al. [70], simulation is an effective educational intervention tool that helps nursing students develop physical and patient skills as well as empathic skills. In particular, Ward et al. [71] highlight the usefulness of virtual simulation as a valuable tool for the empathic development of physiotherapy students, as demonstrated in a longitudinal study.

Sung and Kweon [72] reported, in a quasi-experimental pilot study among nursing students, that those who participated in activities to improve empathic communication had superior self-esteem, empathic ability, interpersonal relationships, and communicative competence scores than those who did not participate in the educational intervention. In light of the aforementioned findings, there is a clear need for ongoing and repetitive educational initiatives that can assist nursing students in enhancing their empathy-based interpersonal relationships and communicative competence within the nursing curriculum.

The advent of digital technologies has the potential to influence empathy, particularly in the context of communication. Further study is required to gain a deeper understanding of the empathic development of student nurses, particularly in the digital age. The issue of digital empathy is emerging and, therefore, nursing students need to receive training in digital empathic and communicative skills [66]. Simulation has been identified as an effective tool for the enhancement of empathic development in nursing students [23,24,25,26,27,28,73].

The results demonstrate a clear correlation between a vocation for care and soft skills development, underscoring the necessity for training in these competencies, particularly empathy. As mentioned above, the absence of sensitivity and empathy precludes the formation of a vocation [1]. It is therefore imperative that health science teachers monitor and evaluate the development of soft skills, such as empathy, assertiveness, and vocation, among their students in order to ensure the delivery of better patient care [13,14,16,45,74,75].

### 4.4. Strengths and Limitations

The strengths of this study include the fact that two different student populations, from physiotherapy and nursing, were studied in a similar academic and clinical training context. In addition, more scientific work needs to be conducted on the simultaneous study of soft skills such as empathy and assertiveness in the professional profiles of future nursing and physiotherapy professionals. Studying two populations with different academic backgrounds and clinical roles but sharing a similar context of professional development and soft skills provides a comparative approach that allows us to identify unique and common patterns in the vocation for care, providing a broader picture applicable to other health science professions.

The limitations include the observational and descriptive nature of the study. In addition, as described above, the Vocational Level Rating Scale is designed for nursing. Although it can be applied to physiotherapy and other health professions and provides interesting results, there are still no more specific scales for professions other than for nursing. The instrument used and the content of the questions were very general in terms of patient care professionals so that it could be applied to students of health sciences related to care.

Therefore, further studies, not only observational studies, and the development of scales for assessing the professional vocation of other health science professions in Spanish are needed.

## 5. Conclusions

Nursing and physiotherapy students demonstrate highly acceptable levels of empathy, assertiveness, and vocation for care, with female students scoring higher than males in both degrees. While nursing students show higher scores in vocation for care, physiotherapy students also exhibit more than acceptable levels. This study contributes to understanding soft skills such as empathy, assertiveness, and vocation for care, providing a holistic perspective on the competencies essential for patient-centred care. It addresses a gap in the literature by examining these skills collectively rather than in isolation.

Significant relationships were identified between vocation for care and empathy, as well as between previous work experience and lower scores in empathy and caring. Additionally, lower empathy scores were associated with higher assertiveness and caring scores. These findings highlight the importance of integrating training in empathy, stress management, and effective communication into health education curricula, considering gender differences and the specific needs of nursing and physiotherapy students.

The professional implications of this study are substantial. First, fostering competencies in empathy, assertiveness, and caring can enhance the quality of patient care, promoting more humane, communicative, and patient-centred practices. Second, cultivating a values-based professional approach grounded in ethical and empathic care is essential for educational and healthcare institutions. Future research should focus on longitudinal studies with educational interventions to further explore these relationships and their impact on clinical practice.

## Figures and Tables

**Table 1:** Interpersonal Reactivity Index (IRI) scores for students of nursing and physiotherapy.

			PT_Perspective Taking	FS_Fantasy	EC_Empathic Concern	PD_Personal Distress
			Median/SD	Median/SD	Median/SD	Median/SD
IRI Score (Academic Year)	First Year	Nursing	27.39/4.15	24.03/6.61	29.27/3.46	15.24/4.64
		Physiotherapy	27.77/4.30	24.37/6.39	28.03/4.33	15.80/4.11
	Second Year	Nursing	28.67/3.72	22.93/5.51	27.97/4.55	15.30/5.03
		Physiotherapy	26.76/4.67	24.52/6.39	27.36/3.59	17.67/4.61
	Third Year	Nursing	28.47/4.61	23.59/5.22	29.35/3.32	14.59/3.52
		Physiotherapy	27.42/3.95	23.92/5.75	27.28/3.59	14.86/4.36
	Fourth Year	Nursing	28.23/3.90	26.38/4.91	29.08/3.75	14.31/2.72
		Physiotherapy	27.76/3.82	20.79/4.54	26.53/3.21	16.88/4.10
IRI Score (Gender)	Female	Nursing	28.14/4.09	24.48/5.88	29.28/3.65	15.41/4.41
		Physiotherapy	27.65/3.96	24.63/6.14	28.57/3.14	16.33/4.63
	Male	Nursing	28.00/3.87	20.54/4.18	26.15/4.10	12.54/2.79
		Physiotherapy	27.12/4.42	21.74/5.11	25.60/3.64	16.22/4.09
IRI Score (TOTAL, n = 226)			27.71/4.12	23.60/5.84	27.92/3.81	15.76/4.40
Cronbach’s α			0.70	0.79	0.82	0.69

**Table 2:** Results of the Rathus test for assertiveness (RAS)—Spanish version for students of nursing and physiotherapy.

			Slightly Assertive	Acceptable Assertiveness	Very Assertive	RAS Score Mean/SD
RAS: Students (Academic year)	First Year	Nursing	n = 1 (1.08%)	n = 29 (31.18%)	n = 3 (3.23%)	15.24/4.64
		Physiotherapy	n = 2 (1.50%)	n = 24 (18.05%)	n = 4 (3.01%)	15.80/4.11
	Second Year	Nursing	n = 5 (5.38%)	n = 18 (19.35%)	n = 7 (2.15%)	15.30/5.03
		Physiotherapy	n = 5 (3.76%)	n = 28 (21.05%)	n = 0 (0.0%)	17.67/4.61
	Third Year	Nursing	n = 3 (3.23%)	n = 12 (12.90%)	n = 2 (2.153%)	14.59/3.52
		Physiotherapy	n = 6 (4.51%)	n = 27 (20.30%)	n = 3 (2.26%)	14.86/4.36
	Fourth Year	Nursing	n = 2 (2.15%)	n = 11 (11.83%)	n = 0 (0.0%)	14.31/2.72
		Physiotherapy	n = 7 (5.26%)	n = 23 (17.19%)	n = 4 (3.01%)	16.88/4.10
RAS: Students (Gender)	Female	Nursing	n =11 (11.83%)	n = 60 (64.52%)	n = 9 (9.68%)	15.41/4.41
		Physiotherapy	n = 15 (11.28%)	n = 55 (41.35%)	n = 5 (3.76%)	16.33/4.63
	Male	Nursing	n = 0 (0.0%)	n = 10 (10.75%)	n = 3 (3.23%)	12.54/2.79
		Physiotherapy	n = 5 (3.76%)	n = 47 (35.34%)	n = 6 (4.51%)	16.22/4.09
RAS: Students (TOTAL n = 226)			n = 33 (14.60%)	n = 170 (75.20%)	n = 23 (10.20%)	−2.16/25.09
Cronbach’s α = 0.86						

The percentages shown in “Academic year” and “Gender” refer to the total number of students on the nursing or physiotherapy degree.

**Table 3:** Results of “Vocation of Service for Human Care” for students of nursing and physiotherapy.

			Regular	Good	Vocation Score
Vocation (Academic year)	First Year	Nursing	n = 9 (9.68%)	n = 24 (25.81%)	81.25/5.40
		Physiotherapy	n = 9 (6.77%)	n = 21 (15.79%)	80.70/10.43
	Second Year	Nursing	n = 6 (6.45%)	n = 24 (25.81%)	83.29/5.89
		Physiotherapy	n = 18 (13.53%)	n = 15 (11.28%)	74.41/9.14
	Third Year	Nursing	n = 2 (2.15%)	n = 15 (16.13%)	78.67/8.33
		Physiotherapy	n = 15 (11.28%)	n = 21 (15.79%)	78.91/8.87
	Fourth Year	Nursing	n = 1 (1.08%)	n = 12 (12.90%)	83.69/4.98
		Physiotherapy	n = 13 (9.77%)	n = 21 (15.79%)	79.13/7.29
Vocation (Gender)	Female	Nursing	n = 16 (17.20%)	n = 64 (68.82%)	81.87/7.60
		Physiotherapy	n = 24 (18.05%%)	n = 51 (38.35%)	80.09/9.44
	Male	Nursing	n = 2 (2.15%)	n = 11 (11.83%)	81.20/5.40
		Physiotherapy	n = 31 (23.31%)	n = 27 (20.30%)	75.88/8.24
Vocation (TOTAL, n = 226)			n = 85 (37.60%)	n = 141 (62.40%)	79.37/11.84
Cronbach’s α = 0.88					

The percentages shown in “Academic year” and “Gender” refer to the total number of students on the nursing or physiotherapy degree.

**Table 4:** Correlation analysis—Empathy subscales, assertiveness, and healthcare vocation.

	Empathy (PT)	Empathy (FS)	Empathy (EC)	Empathy (PD)	Assertiveness (RAS)	Healthcare Vocation	HC Vocation Dimension 1	HC Vocation Dimension 2	HC Vocation Dimension 3
Empathy (PT)		0.061 *p* = 0.36	0.306 * p < 0.001	−0.116 *p* = 0.08	−0.004 *p* = 0.95	0.252 * p < 0.001	0.289 * p < 0.001	0.153 *p* = 0.02	0.183 *p* = 0.006
Empathy (FS)			0.442 * p < 0.001	0.202 *p* = 0.002	−0.096 *p* = 0.15	0.061 *p* = 0.37	0.118 *p* = 0.08	−0.028 *p* = 0.68	0.083 *p* = 0.21
Empathy (EC)				0.160 *p* = 0.016	−0.132 *p* = 0-047	0.351 * p < 0.001	0.461 * p < 0.001	0.119 *p* = 0.07	0.346 * p < 0.001
Empathy (PD)					−0.317 * p < 0.001	−0.230 *p* = 0.001	−0.143 *p* = 0.031	−0.260 * p < 0.001	−0.148 *p* = 0.026
Assertiveness (RAS)						0.141 *p* = 0.035	0.058 *p* = 0.39	0.230 *p* < 0.001	0.013 *p* = 0.85
Healthcare Vocation							0.841 * p < 0.001	0.865 * p < 0.001	0.794 * p < 0.001
HC Vocation Dimension 1								0.529 * p < 0.001	0.577 * p < 0.001
HC Vocation Dimension 2									0.553 * p < 0.001
HC Vocation Dimension 3									

Abbreviations: EC = empathic concern; FS = fantasy; PD = personal distress; PT = perspective taking; RAS = Rathus Assertiveness Scale; HC = healthcare. * Correlations higher than 0.250.

## Data Availability

The data supporting the findings of the study are available from the corresponding author upon reasonable request. The data are not publicly available due to privacy or ethical restrictions.

## Public Involvement Statement

There was no public involvement in any aspect of this research.

## Guidelines and Standards Statement

This manuscript was drafted against the Strengthening the Reporting of Observational Studies in Epidemiology (STROBE) Statement.
